# Gross Hematuria in an Elderly Smoker Male Due to Eosinophilic Cystitis

**DOI:** 10.7759/cureus.12400

**Published:** 2020-12-31

**Authors:** Ali F Al Sbihi, Nouraldeen Manasrah, Sarah Al Qasem, Mazen Abdelhady, Dongping Shi

**Affiliations:** 1 Internal Medicine, Detroit Medical Center Sinai Grace Hospital, Detroit, USA; 2 General Practice, Luzima Hospital, Irbid, JOR; 3 Urology, Detroit Medical Center Sinai Grace Hospital, Detroit, USA; 4 Pathology, Detroit Medical Center Sinai Grace Hospital, Detroit, USA

**Keywords:** hematuria, eosinophilic cystitis, bladder

## Abstract

This is a case of a 71-year-old smoker man who presented with four days of gross hematuria, which turned to be caused by eosinophilic cystitis (EC) proven by bladder biopsy. EC is a rare clinical and pathological inflammatory condition of the bladder with an unknown exact cause. It can present with hematuria, urinary frequency, dysuria, and suprapubic pain. Sometimes, the presentation can mimic urinary tract infection (UTI) or malignancy, especially in older patients.

## Introduction

EC was first described by Brown and Palubinskas in 1960 [1, 2]. This inflammation is characterized by transmural involvement of the bladder predominantly with eosinophils. It is associated with fibrosis with or without muscle necrosis. It fairly affects adult men and women, although a very minimal male predominance is seen among the pediatric age group [3]. EC has associations with numerous factors, such as allergy, bladder tumor, bladder trauma, parasitic infections, and chemotherapeutic agents with no clear cause for it [4]. This case report discusses the presentation, diagnosis, and management of EC.

## Case presentation

A 71-year-old male with a history of stroke, peripheral vascular disease, and polysubstance abuse was admitted to the hospital with gross hematuria for four days. The patient was also complaining of mild pain with urination. He denied any difficulty urinating, flank pain, suprapubic pain, abdominal pain, other urinary symptoms, or weight loss. He also denied other sources of bleeding, recent trauma, recent drug, or alcohol use. He is not sexually active. He is a 25-pack year cigarette smoker, but he had recently quit. Medications include baby aspirin and rosuvastatin. There was no family history of similar presentations.

On presentation, his blood pressure was 159/66 mmHg, heart rate was 78 beats/minute, respiratory rate was 17 breaths/minute, oxygen saturation was 96% on room air, and he had a temperature of 36.6 Celsius. Physical examination revealed circumcised phallus, urethral meatus in orthotopic position, and bilateral descended testicles. There was no ecchymosis or signs of external trauma to the scrotum, perineum, or penis. The bladder was palpable. There was no crepitus, erythema, or warmth over the scrotum. The abdomen was soft, non-tender, and non-distended.

Laboratory findings showed hemoglobin of 8.4 g/dl (baseline: 10-11 g/dl; normal:14-17). White blood cell (WBC) count was 7300 cells/mm^3^ (4000-11,000 cells/mm^3^) and absolute eosinophil count was 0.2 cell/mm^3^, it was up to 700-800 cell/mm^3^ in the last three months (normal: 0-500 cell/mm^3^). Mean corpuscular volume (MCV) was 89.6 femtoliter (80-100 femtoliter), red cell distribution width (RDW) was 13.7% (11.8- 14.5%), prothrombin time (PT) was 10.3 seconds (11-13.5 seconds), international normalized ratio (INR) was 0.96 (0.9-1.1), and platelet count was 281,000/mm^3^ (150,000-400,000/mm^3^). Electrolytes were within normal limits.

Urinalysis showed cloudy red urine, red blood cell (RBC) count was more than 100 cell/high power field (HPF) (0-4 cell/HPF), WBC count was less than 5 cell/HPF (2-5 cell/HPF), squamous cells were less than 5 cell/HFP (0-5 cell/HPF), urine blood was 3+, urine protein was 3+, urine pH was 7.5 (4.6-8.0), and there were 2+ bacteria. Bilirubin, urobilinogen, leukocyte esterase, casts, crystals, and nitrite were negative. Urine drug screening was negative.

**Figure 1:**
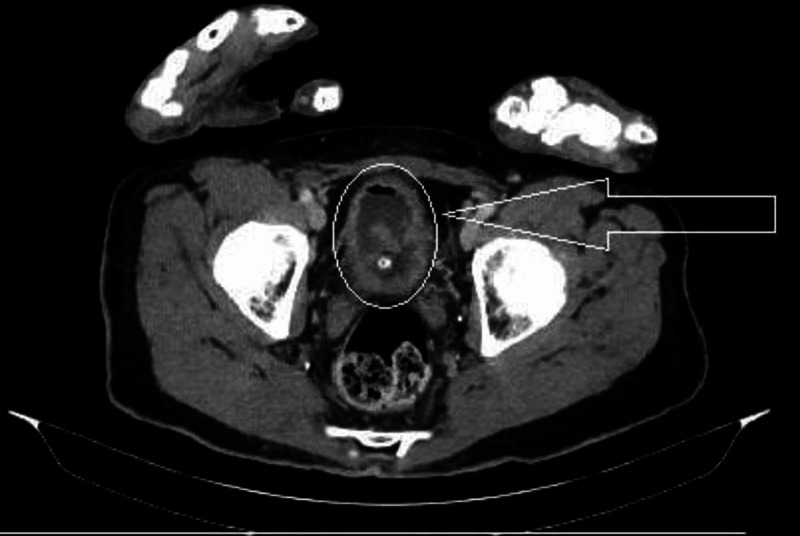
CT of abdomen-pelvis showed thickened bladder wall and polypoid projections into the bladder’s lumen

Pelvis X-ray showed no acute fractures or dislocation, kidneys-ureters-bladder (KUB) X-ray showed no evidence of stones or injuries. Computed tomography (CT) scan of the abdomen and pelvis with contrast showed diffuse nodular thickening of the bladder wall with multiple polypoid projections into the lumen, which were concerning for malignancy with interval development of mild hydroureter within the distal right ureter without hydronephrosis (Figure 1). The patient was given one-liter intravenous fluids (IVF) bolus, and a 1g IV ceftriaxone course was started.

Urology was consulted who performed cystoscopy, clot evacuation, bladder biopsy fulguration, and bilateral retrograde pyelogram. The latter study was unremarkable. Foley’s catheter was inserted, and IVF continued. Urine culture came negative for growth, urine started to gradually clear up, and the antibiotics course was stopped after five days.

**Figure 2:**
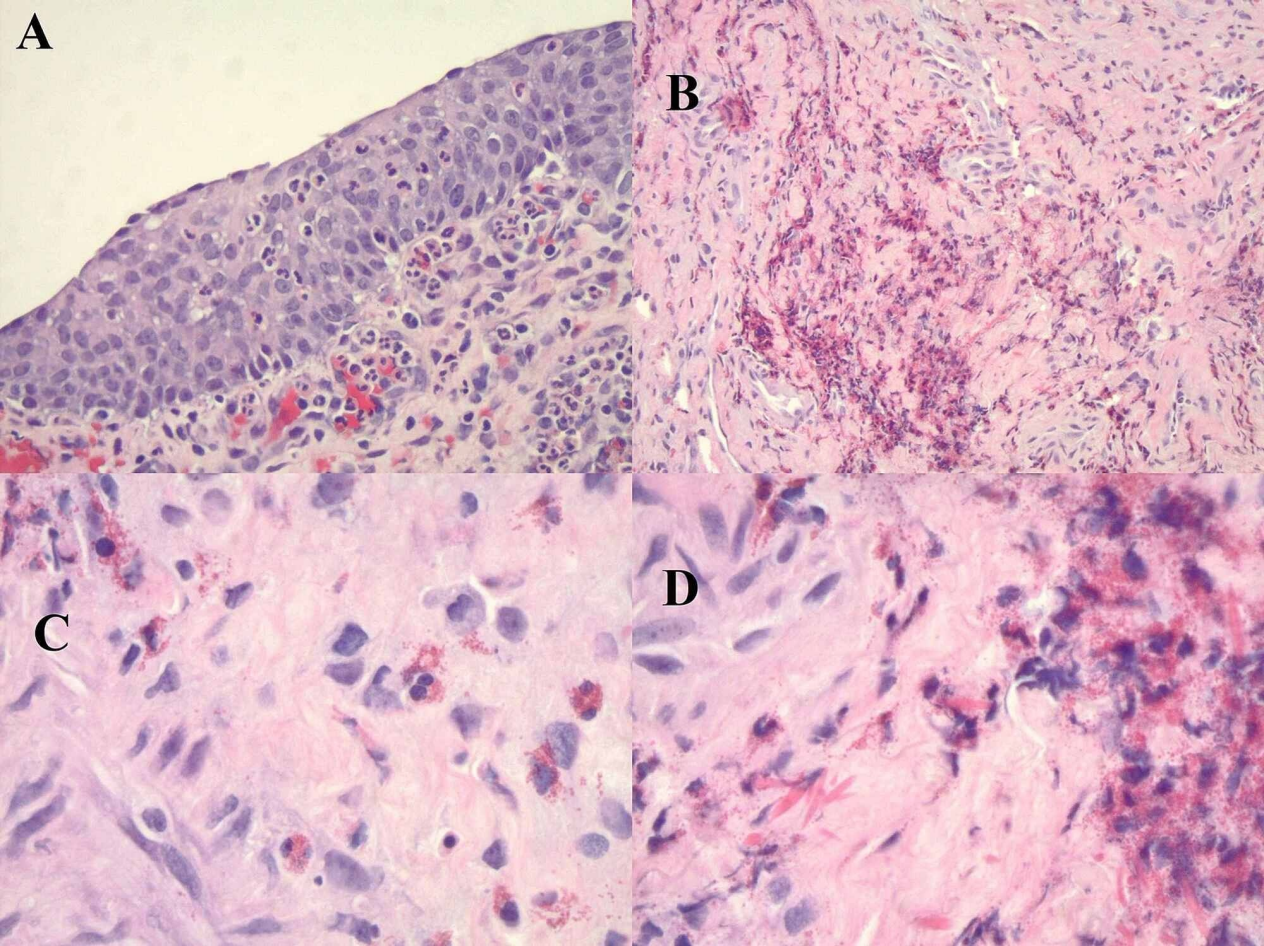
Bladder biopsy Image A shows acute cystitis with eosinophilic infiltrates; image B shows increased eosinophils; image C is a high power field view showing eosinophilic infiltrates; image D shows Charcot-Leyden crystals. Yamada and Taguchi criteria are 20 or more eosinophils per five 20x fields with edema and occasional muscle necrosis with possible Charcot-Leyden crystals [5].

Bladder cancer was highly suspected, and then interestingly, the bladder biopsy showed acute phase eosinophilic cystitis in the posterior and right lateral walls with prominent submucosal hemorrhage in the right and left lateral walls (Figure 2). The biopsy was negative for carcinoma. Foley’s catheter was maintained as an inpatient; the hospital course continued to be uncomplicated. The patient continued close outpatient follow-up with no recurrence of symptoms or microscopic hematuria, as proven by urine analysis. The patient did not require any further management except for close follow-up.

## Discussion

EC is a bladder inflammation that is primarily caused by eosinophils. It can mimic UTI or malignancy by its presentation. The most common symptoms of EC are frequency, hematuria, dysuria, and suprapubic pain. Less common ones include nocturia and urinary retention, with the latter being more frequently reported in women and children. Proteinuria, microscopic/macroscopic hematuria, and peripheral eosinophilia are found only in a small percentage of patients [3, 6]. Cystoscopy with biopsy is the gold standard test for diagnosis.

Hellstrom et al. described that EC occurs either in patients with allergy, asthma, atopy, and peripheral eosinophilia, it is commonly seen in women and children or in patients with a history of bladder trauma; this tends to occur in elderly men with a history of surgery for benign prostatic hyperplasia, open bladder surgery or resection of bladder tumor [4]. EC is likely caused by the antigen-antibody reaction, leading to the production of numerous immunoglobulins that cause eosinophils’ activation and initiate the inflammatory process. Due to reported cases of eosinophilic cystitis in patients with celiac disease, an underlying malfunctioning of the immune system has also been proposed [7].

Urinalysis may show proteinuria and microscopic hematuria; however, urine cultures are usually negative [3]. Eosinophiluria is rare due to many factors, including prompt degradation of eosinophils, minimal mucosal shedding from the urothelium, and that eosinophilia is present in other renal and urological conditions, so the presence of eosinophiluria is not diagnostic [8]. In blood tests, eosinophilia may be useful; however, it is found in approximately 50% of patients with a history of allergy or atopy [9].

On imaging, varying degrees of bladder wall thickening may be found, from diffuse thickening to mass formation, and that depends on the stage of EC [10]. Hydronephrosis with bladder and ureteral filling defects were noted in some studies [6, 8].

A biopsy is the gold standard test for the diagnosis of EC; it usually shows a transmural inflammation of eosinophils, mainly in the lamina propria. EC can be classified as acute or chronic, based on histology. Tissue eosinophilia, mucosal edema, hyperemia, and muscle necrosis are more commonly found in acute phases. Chronically, scarring can be clearly noticed, but tissue eosinophilia is not common [10, 11].

**Figure 3:**
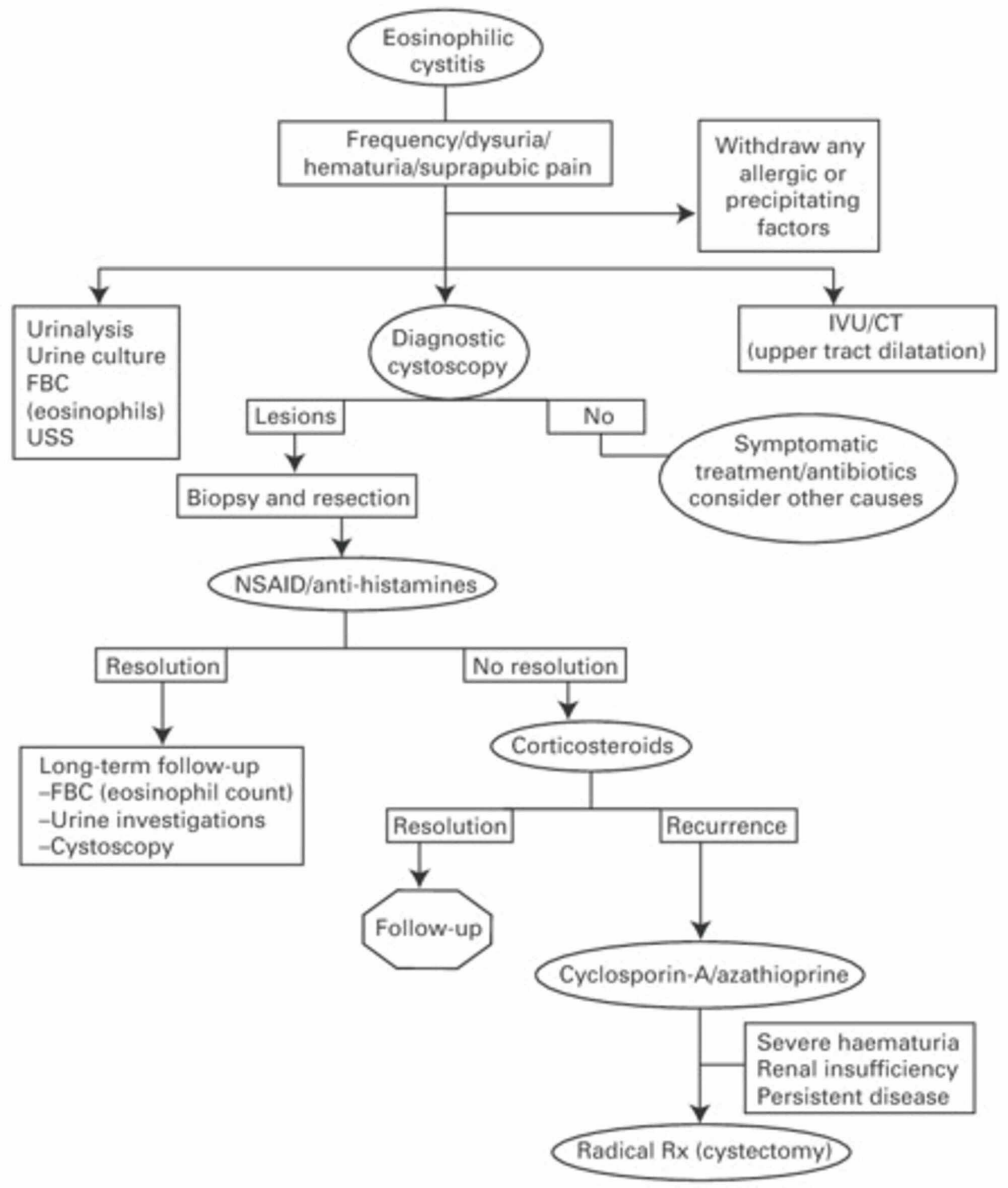
Algorithm for EC treatment The permission to republish the image is obtained from the original publisher. Source: [11] EC - eosinophilic cystitis; IVU - intravenous urogram; FBC - full blood count; USS - ultrasound scan; NSAID - nonsteroidal anti-inflammatory drug; Rx - treatment

UTI or medications like tranilast and mitomycin C must be identified as precipitating factors [8]. Afterward, nonsteroidal anti-inflammatory drugs (NSAIDs) or antihistamines are the first-line medications for the management of EC, corticosteroids are the second line, with azathioprine and cyclosporine-A come as the third line. After medical management failure, operative intervention should be considered like diathermy, lesion resection, or cystectomy. Algorithms for EC management have been suggested as there are no standards for treatment. One example of these algorithms is shown (Figure 3) [11]. Our patient did not require any medical management because the cystoscopy procedure was diagnostic and therapeutic at the same time.

Prognosis is usually benign with complete resolution in most affected patients. Long term monitoring by blood tests, urine analysis, and imaging is crucial. Sometimes, cystoscopy may be indicated to avoid renal and bladder damage and to rule out bladder cancer. Because lesions tend to recur despite the above therapy, long‐term follow‐up is mandatory [11, 12].

## Conclusions

EC is a rare inflammatory condition of unknown cause that mimics other urological conditions. A biopsy is the gold standard test for the diagnosis. EC is best managed by a combination of oral medical therapy and surgical intervention. Although most patients are cured, recurrence is common, so follow up after diagnosis and treatment is mandatory.
